# Engineering Crack Formation in Carbon Nanotube-Silver Nanoparticle Composite Films for Sensitive and Durable Piezoresistive Sensors

**DOI:** 10.1186/s11671-016-1626-z

**Published:** 2016-09-22

**Authors:** Phong Tran Hoang, Nicolas Salazar, Thomas Nolan Porkka, Kunal Joshi, Tao Liu, Tarik J. Dickens, Zhibin Yu

**Affiliations:** Department of Industrial and Manufacturing Engineering, High-Performance Materials Institute, Florida State University, 2005 Levy Ave., Tallahassee, FL 32310 USA

**Keywords:** Piezoresistive, Strain sensors, Carbon nanotubes, Silver nanoparticles, Composites, Crack

## Abstract

We report highly sensitive and reliable strain sensors based on silver nanoparticle (AgNP) and carbon nanotube (CNT) composite thin films. The CNT/AgNP was prepared by a screen printing process using a mixture of a CNT paste and an AgNP ink. It is discovered that the sensitivity of such sensors are highly dependent on the crack formation in the composites. By altering the substrate use and the relative ratios of AgNPs and CNTs, the formation and propagation of cracks can be properly engineered, leading to piezoresistive strain sensors with enhanced sensitivity and robustness.

**Electronic supplementary material:**

The online version of this article (doi:10.1186/s11671-016-1626-z) contains supplementary material, which is available to authorized users.

## Background

Strain gauges are of interest to many applications including structural health monitoring and active input control devices [1–6]. Current commercial strain gauges are dominated by semiconducting silicon crystal gauges and metallic foil gauges. Semiconductor-based strain gauges offer high sensitivity (gauge factor (GF) of 100–170) [7] but are quite rigid and too fragile for dynamic applications. Metallic foil gauges are more robust but lack sensitivity (GF of 2–5) [8]. Recently, a variety of nanomaterials such as graphene, carbon nanotubes (CNTs), metallic nanoparticles (NPs), and metal nanowires have shown promising properties for piezoresistive strain sensors [1, 9–14]. Many of those nanomaterials can be processed using solution based methods, potentially enabling low-cost fabrication and large area application.

Individual single-walled CNT (SWCNT) has demonstrated exceptional piezoresistive sensitivity with a remarkable high gauge factor (GF) of up to ~2900 [15–19]. However, it is a great challenge to obtain the intrinsic piezoresitive sensitivity of individual SWCNTs for large area practical applications. The GFs were found to drop below 1.0 when CNT yarns or films were used [20, 21]. Mixing the CNTs with a polymer to form a composite can also increase the GFs; however, this process sacrifices the high conductivity of the CNTs leading to inefficient sensors [22, 23].

In contrast to CNTs, strain sensors based on conductive NP films have been demonstrated to possess much higher GFs. For instance, Farcau et al. [24, 25] developed strain gauges fabricated from monolayers and multilayers of wire-patterned AuNPs. They reported GFs in the range of 59–135. Herrmann et al. obtained strain gauges with GFs between 50 and 200 using polymer crosslinked AuNPs [26]. Radha and co-workers reported GFs up to 390 based on micromolded Pd-nanoparticle-Carbon μ-stripes [27]. Kang et al. sputtered a platinum NP thin film on a polyurethane acrylate elastomer substrate. They realized strain gauges with GFs over 16,000 [28]. These NP films form small cracks under tension; and the disconnection–reconnection of the cracks becomes the driving mechanism of their resistance changes with strain. However, this crack formation can cause such sensors to malfunction after a limited number of strain cycles as the NPs separate further and further due to creep of the host substrates and the nanoparticle films.

Herein, we fabricated silver NP (AgNP) and CNT composite thin films from a screen printing process. Such sensors have recently been reported [29, 30]; nonetheless, the underlying mechanism has not been studied. In this work, we discovered that by altering the substrates and the relative ratios of AgNPs and CNTs, we can tune the formation and propagation of cracks within the composites, leading to piezoresistive strain sensors with enhanced sensitivity and robustness.

## Methods

### Fabrication of CNT/AgNP Piezoresistive Sensors

**Fig. 1:**
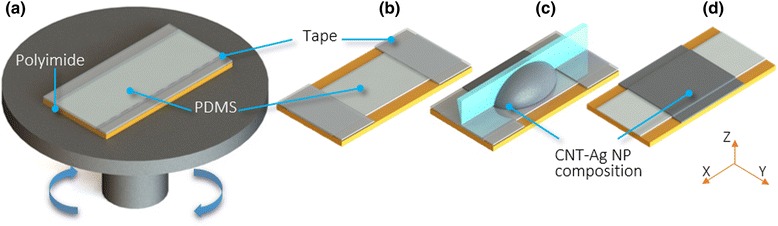
A schematic of the CNT-AgNP thin film fabrication process

The CNT/Ag piezoresistive sensors were fabricated according to the scheme shown in Fig. 1, which involves polyimide (PI)/polydimethylsiloxane (PDMS) bilayer substrate preparation and subsequent deposition of the CNT/AgNP composite film. To prepare the PI/PDMS substrate, a PI film 127 μm thick (2271K3, McMaster-Carr) was cleaned thoroughly with soap, water, and acetone. Subsequent to the cleaning, the PI substrate was dried at room temperature and treated with oxygen plasma at 100 W power and a flow rate of 100 sccm for 5 min (CUTE-MPR, Femto Science). As illustrated in Fig. 1a, a mixture of PDMS base and curing agent with 10:1 weight ratio (Sylgard 184, Dow Corning) was cast onto the PI substrate for thin film formation via spin coating at 6000 rpm. The wet film was cured in an oven at 100 °C for ~2 h. The cured PDMS was about 20 μm thick (Fig. 1b). It was then treated with oxygen plasma (100 W, 100 sccm O_2_, 5 min) again to allow for enhanced adhesion with the subsequently deposited CNT–AgNP composite layer. The CNT–AgNP composite paste was prepared by thoroughly mixing a AgNP ink (Paru Company, Ltd., 42 wt.%) and a CNT paste (SWeNT Inc.; the SWCNTs were dispersed in volatile solvents without a polymer binder with a concentration of 0.2 wt.%) at varied mass ratios and then spread onto the plasma-treated PDMS side via screen printing (Fig. 1c). A final thermal annealing treatment that was performed at 100 °C for 12 h was applied to remove the residual solvents and complete the CNT/AgNP piezoresistive sensors (Fig. 1d).

The concentration of the AgNP ink and the CNT paste were both verified by thermogravimetric analysis (TGA Q50, TA Instruments) as shown in Additional file 1: Figure S1a, b in the supporting information. Although all the solvent residuals are removed from the composites after annealing process, for simplicity, we used the concentration ratios of the CNT paste and the AgNP ink to name the composite films. The real concentration ratios of CNT and AgNPs in the final composites were calculated as shown in Additional file 2: Table S1 in the supporting information. A CNT/Ag piezoresistive sensor directly deposited onto a PI substrate was also prepared for comparison and examining the effect of PDMS layer. During the CNT/AgNP piezoresistive sensor preparation process, scotch tapes were applied to define the size of the PDMS as well as the size and thickness of CNT/AgNP sensing area. It is important to note the plasma treatment of PDMS is crucial for enhancing adhesion between the CNT-AgNP layer and the PDMS substrate. Without such a treatment, the formed thin film can be easily peeled off and run-to-run measurement error becomes significant.

### Piezoresistivity Evaluation and Characterization of CNT-AgNP Films

The CNT-AgNP thin films were cut into strips of size L × W = 1.2 × 0.2 cm used for piezoresistive performance evaluation, which was carried out through a coupled electrical–mechanical testing. In brief, the CNT-AgNP thin film strip with silver paste (Silver Conductive Paint, 503, Electron Microscopy Sciences) applied on both ends was clamped into a dynamic mechanical analyzer (DMA) (Q800, TA instruments). The testing gauge length was set at 1 cm. The sample was then subjected to a sinusoidal cyclic strain of amplitude 0.4 % at a rate of 4 %/min. In the meantime, a Keithley 2401 Source Meter controlled by a homemade LABVIEW user interface was used to record the sample’s electrical resistance. According to the recorded electrical resistance, the sheet resistance of the thin film sample – R_s_ was calculated by R_s_ = R × (W/L). More information on the coupled electrical–mechanical testing protocol and apparatus can be found in our previous work [31, 32]

## Results and Discussion

**Fig. 2:**
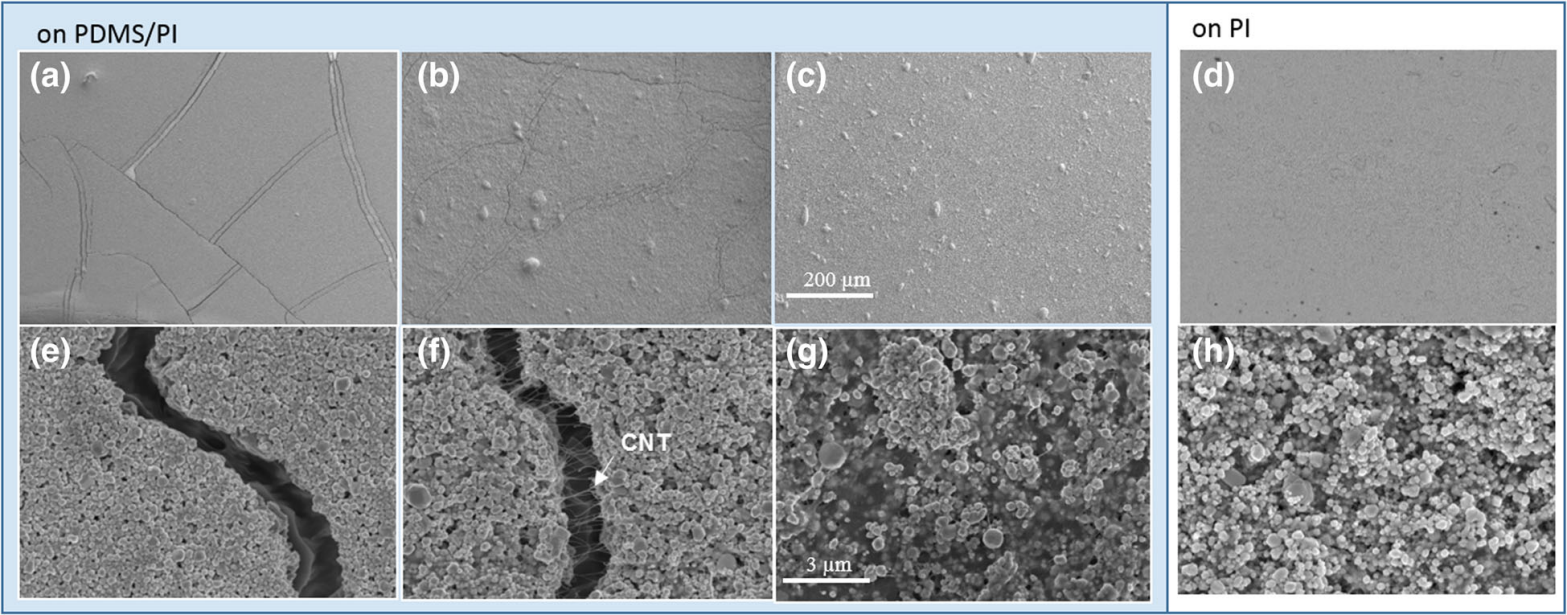
The SEM images of the CNT-AgNP thin films with CNT loading at 0 wt.% (**a**, **e**), 70 wt.% (**b**, **f**), 95 wt.% (**c**, **g**) deposited on PDMS/PI substrate, and 70 wt.% (**d**, **h**) deposited on PI substrate. *Scale bar* in **a**–**d** is 200 μm and in **e**–**h** is 3 μm

Scanning electron microscopy (SEM) was performed with field-emission SEM (FEI Nova 400) at 5 kV for examining the morphologies of CNT-AgNP thin films. CNT-AgNP thin films with varied CNT loadings were prepared according to the procedure in Fig. 1. Figure 2a–c shows the low magnification SEM images of composite film deposited on PDMS/PI substrates with CNT loadings at 0, 70, and 95 wt.%, respectively. The corresponding high-magnification images are shown in Fig. 2e–g.

Without CNTs, the AgNPs formed cracks on top of PDMS/PI (Fig. 1a, e). This is likely due to thermal expansion mismatch between the AgNP film and the PDMS. Since the composite films were casted from a solution and dried at 100 °C, cracks can form during the solvent evaporation and cooling steps. As seen from Fig. 1a, the cracks were fairly straight and extended hundreds of micrometers long, suggesting a brittle fracture behavior in such a film. The film was nearly non-conductive, indicating the cracks had spread throughout the film. Such a brittleness feature can be found in all composites with CNT loading less than 50 wt.%.

At 70 wt.% CNT (Fig. 2b, f), the average width of the cracks was drastically reduced, and the crack propagation became more twisted. This observation suggests the composite became more ductile after blending with CNTs. This can be understood from the reinforcement effect of the CNTs, which mitigated and altered the crack propagation. In contrast to the film without CNTs, the gaps in the cracked region were connected by threads of CNTs in the AgNP-CNT composite film. The authors hypothesize the CNT bridges helped maintain the conductivity of the composition film regardless crack formation and propagation. With a 95 % CNT paste concentration (Fig. 1c, g), cracks nearly disappeared in the composite film, further proving the reinforcement characteristic of the CNTs. The effect of substrate was also investigated for crack formation in the composite films. Figure 2d, h shows the SEM images of samples with 70 % CNT paste on a PI substrate. No crack formation was seen. It is expected since the thermal expansion coefficient of the PI is a lot smaller than the PDMS and the thermally induced stress is minimized at the AgNP-CNT and substrate interface.

**Fig. 3:**
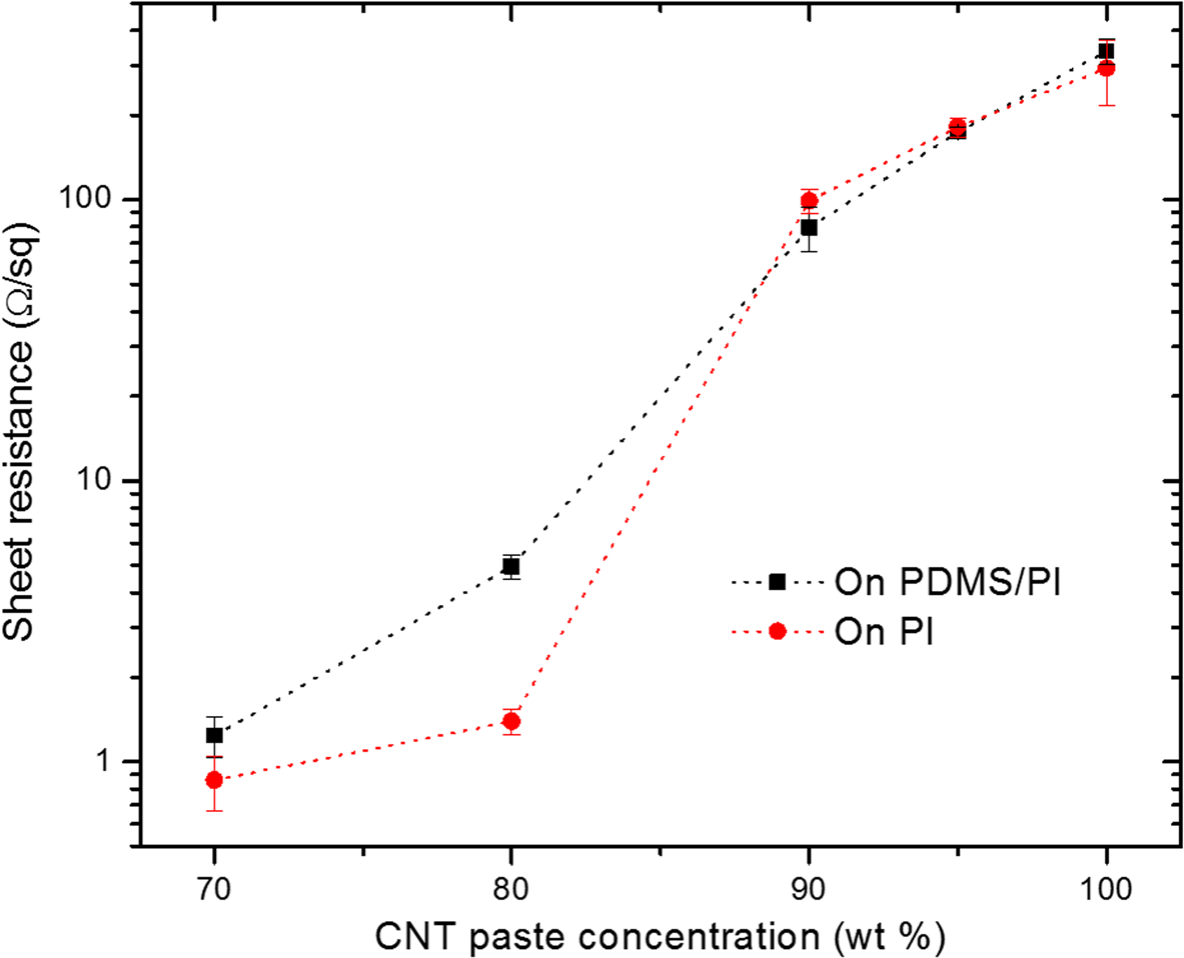
Sheet resistance of CNT-AgNP composites at different CNT paste concentration on PDMS/PI and PI substrate

The sheet resistance of the CNT-AgNP thin films were measured with CNT paste concentrations varying from 70 to 100 wt.% on both PDMS/PI and PI substrates. The results are shown in Fig. 3. Overall, the sheet resistance of the thin films increases with increasing CNT concentration. This is expected since the CNTs exhibit a lower electrical conductivity than AgNP aggregates [26]. At 70 and 80 % CNT paste concentrations, the composite films showed higher resistance values on the PDMS/PI substrate. With a CNT paste concentration at above 90 %, the resistance became nearly comparable on both substrates. Such results are in good agreement with the morphology observation as cracks form in the samples with lower CNT concentrations, but higher CNT concentrations provide mechanical reinforcement so cracks do not form regardless of substrate as shown in Fig. 2b, f, c, g.

The sheet resistance on both substrates shows an abrupt increase at a threshold CNT paste concentration between 80 and 90 %, indicating an evolution of electrical flow pathway in the composite films. It is conjectured that at lower CNT paste concentrations (≤80 %), the AgNPs can maintain long range connectivity and conduct a majority of the current flow; however, increasing the CNT paste concentration to ≥90 % causes the AgNP connection to become interrupted; thus, the current flow has to pass through the higher resistance CNTs. It is interesting to note this threshold concentration (80–90 %) correlates well with the CNT paste concentration upper limit (95 %) for crack formation on the PDMS/PI substrate, suggesting the same origin of CNT distribution to affect the electrical current transportation and mechanic reinforcement in the composite thin films. To this end, we have achieved AgNP-CNT composite films with tunable cracking features through substrates with different thermal properties, as well as engineering the relative ratios of the AgNPs and CNTs.

**Fig. 4:**
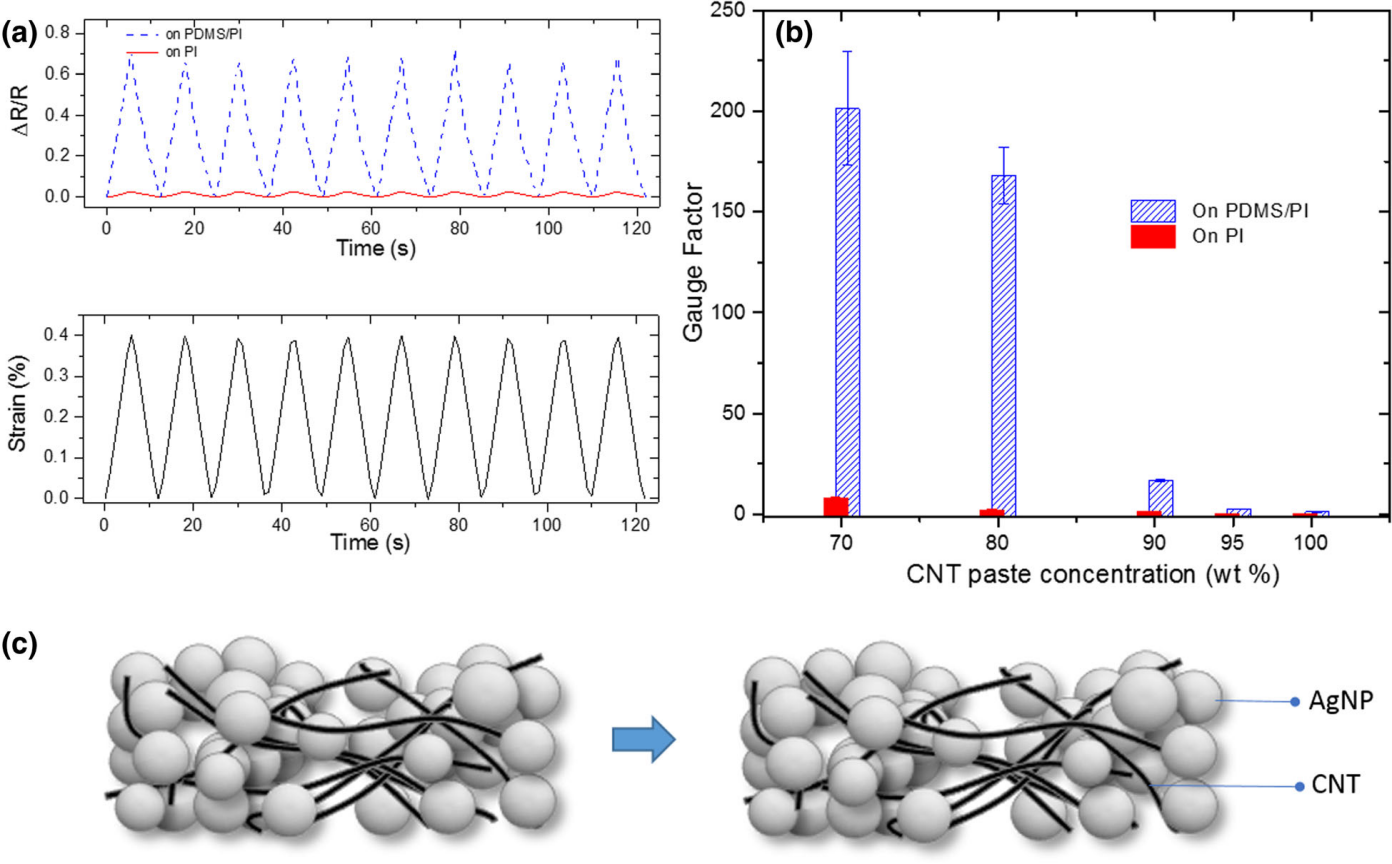
*Top*: evaluation of the electrical resistance and piezoresistive behavior of the CNT-AgNP thin films. **a** Piezoresistive response of the wt.% 70 samples with and without PDMS. **b** Gauge factors of CNT-AgNP sensor at different CNT paste concentration with and without PDMS. **c** The schematic illustration of the mechanism of piezoresistive behavior of CNT-AgNP thin film

With the coupled electrical–mechanical testing procedure, we evaluated the composites’ piezoresistive behaviors. Figure 4a compares the relative resistance change ΔR/R of the 70 wt.% CNT paste sample respectively deposited on PDMS/PI and PI substrate when subjected to a cyclic tensile strain with a maximum amplitude set at *ε* = 0.4 %. The sample deposited on the PDMS/PI substrate showed a significantly larger resistance change (~22× greater) compared to the film on the PI substrate. The same measurement was carried out using films with other compositions and gauge factors were calculated. GFs were calculated according to Eq. 1:

1 $$ \mathrm{Gauge}\ \mathrm{factor}\ \left(\mathrm{G}\mathrm{F}\right)=\frac{\Delta R}{R\varepsilon } $$

Figure 4b compares the GFs for all the films with various CNT concentrations on PDMS/PI or PI substrates. GFs of 221.2 and 168.1 were obtained for 70 wt.% CNT and for 80 wt.% CNT paste on PDMS/PI substrates, respectively. In addition, the GF continued to decrease as the CNT wt.% increased. For instance, a GF of 16.6 was achieved for the 90 %, 2.5 for the 95 %, and 1.3 for the 100 % CNT paste composites on PDMS/PI substrates. In contrast, the films on PI substrates showed fairly low GFs for all CNT paste concentrations from 70 to 100 wt.%. It was observed that the high GFs were achieved only for samples with cracks suggesting they play an integral role in resistance change.

Crack-based strain sensors have been previously reported based on sputtered metal thin films on elastomeric substrates [28, 33]. It is speculated that the observed strain sensitivity is caused by separating the AgNPs upon stretching as shown in Fig. 4c. The current flow path is then changed from low resistance AgNP-AgNP dominant paths to high resistance AgNP-CNT-AgNP ones. The existence of cracks is essential for high sensitivity, since the applied stress will be concentrated around the cracks amplifying the stress induced strain response. Such an amplification effect does not exist in uniform samples without cracks because the stress is applied homogenously throughout the film as opposed to being concentrated at the cracks. This explains the low sensitivity in composite films on PI substrates and films on PDMS/PI substrates with a high CNT concentration.

**Fig. 5:**
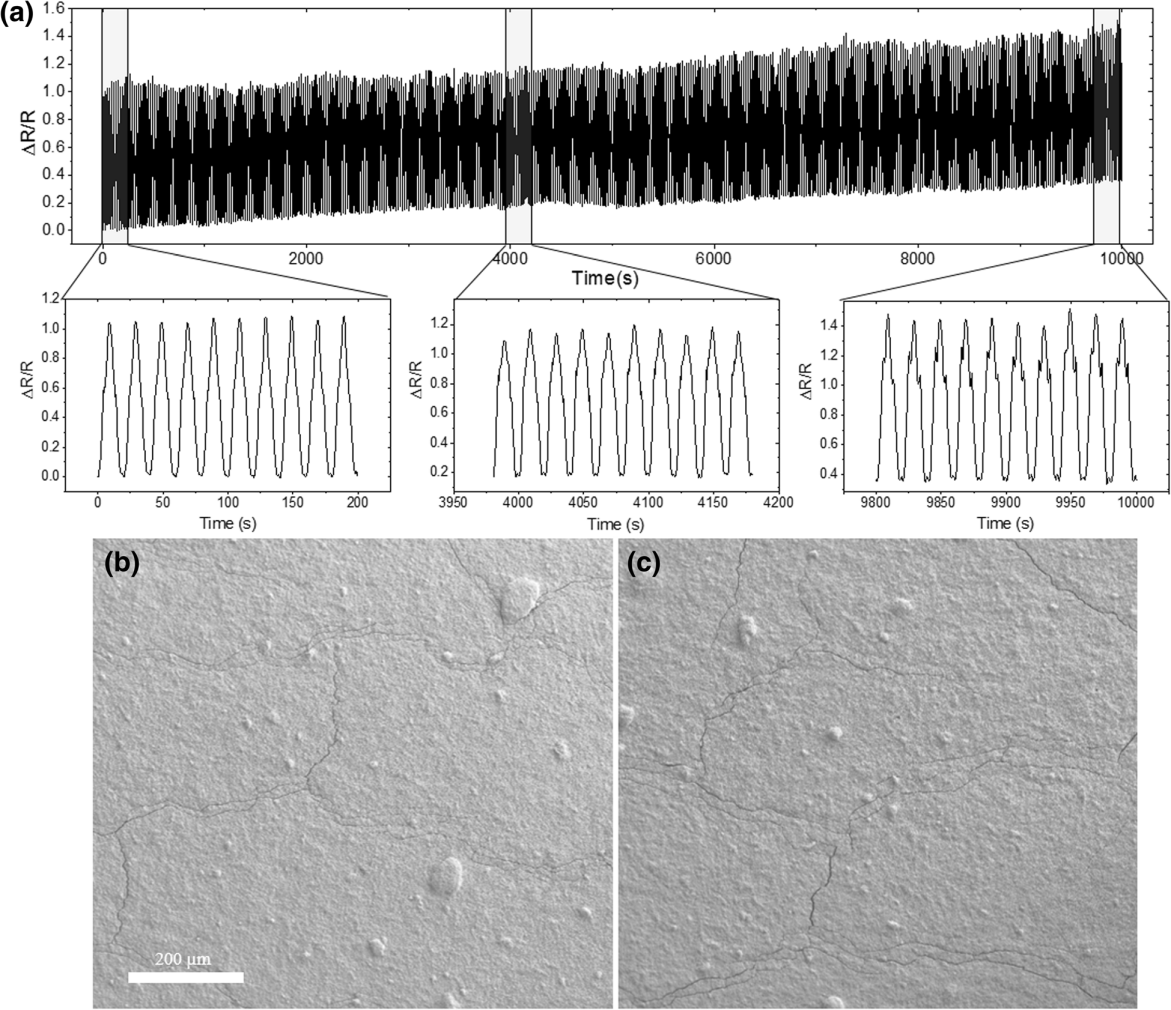
**a** The piezoresistive response of the CNT-AgNP thin film deposited on PDMS/PI with CNT loading of 70 wt.% under 500 cycles of a sinusoidal tensile test (0.5 % in amplitude, 0.05 Hz in frequency). **b**, **c** The surface morphologies of the same sample by SEM imaging before and after 500 cycles

The strategy of using AgNP/CNT composites could potentially enable very robust and durable strain sensors. In previous designs by others, using cracked metallic nanoparticles [34, 35] provided low durability sensors. This is because the particles around the cracks drifted further apart due to inevitable creep behaviors of the substrates and the nanoparticle films, leading to permanent loss of electrical conductivity and failure of the sensors. By incorporating CNTs, the cracks will always be bridged by threads of CNTs, therefore maintaining the electrical conductivity and functionality of such sensors, and would allow for extended lifespans. Mechanical robustness of the CNT-AgNP thin film sensors was evaluated as shown in Fig. 5a. In the test, a sinusoidal strain with a maximum amplitude of 0.5 % and oscillation frequency of 0.05 Hz was used. A total of 500 test cycles were carried out. The base resistance of the film at zero strain exhibited an increasing tendency with cycles, and a 40 % increase was observed after the 500 test cycles. Such a behavior can be accounted for by the creeping of the PDMS substrate and loss of some AgNP conductive pathways. Nonetheless, the resistance changes for each test cycle remained fairly constant, proving the robustness of the composite samples. We assume the sensors can be pre-calibrated to compensate for the base resistance shift due to material creep. The morphology of the composite before and after the 500 cycles of stress test was examined by SEM as shown in Fig. 5b, c. No obvious broadening of the cracks was detected after the 500 cycles, supporting the mechanical reinforcement of the incorporated CNTs in the composites.

## Conclusions

CNT-AgNP composite thin films were fabricated using a solution-based screen printing process. It was discovered that the substrate and the CNT concentration both played important roles in developing microscopic cracks in the composite films. The crack formation leads to piezoresistive sensors with high GFs up to 221.2. The incorporation of CNTs has also been shown to reinforce the composite film, resulting in durable sensors that can survive 500 stretching-releasing cycles (0.5 % maximum strain) without sensitivity degradation. By controlling the concentration ratio of CNTs to AgNPs in the nanocomposites and the supporting substrates, we were able to engineer the crack formation to achieve stable and high-sensitivity sensors. Also, with a simple, low-cost, and easy to scale up fabrication process, this may find use as an alternative to traditional strain sensors.

## Additional Files

Additional file 1:
**Figure S1.** Thermogravimetric analysis (TGA) in nitrogen of (a) CNT paste (CNT wt.% ~0.2) and (b) AgNPs ink (AgNPs wt.% ~42). (TIF 130 kb) Additional file 2:
**Table S1.** The weight percentage of CNTs and AgNPs in the composite at different CNT paste concentrations. (XLSX 9 kb)

## Abbreviations

AgNP: Silver nanoparticle
CNT: Carbon nanotube
DMA: Dynamic mechanical analyzer
NP: Nanoparticle
PDMS: Polydimethylsiloxane
PI: Polyimide
SEM: Scanning electron microscopy
TGA: Thermogravimetric analysis
