# Supplementary figures and images for: Engineering Crack Formation in Carbon Nanotube-Silver Nanoparticle Composite Films for Sensitive and Durable Piezoresistive Sensors
Source: Nanoscale Res Lett. 2016 Sep 22;11:422. doi: 10.1186/s11671-016-1626-z (PMC5033798; doi:10.1186/s11671-016-1626-z)

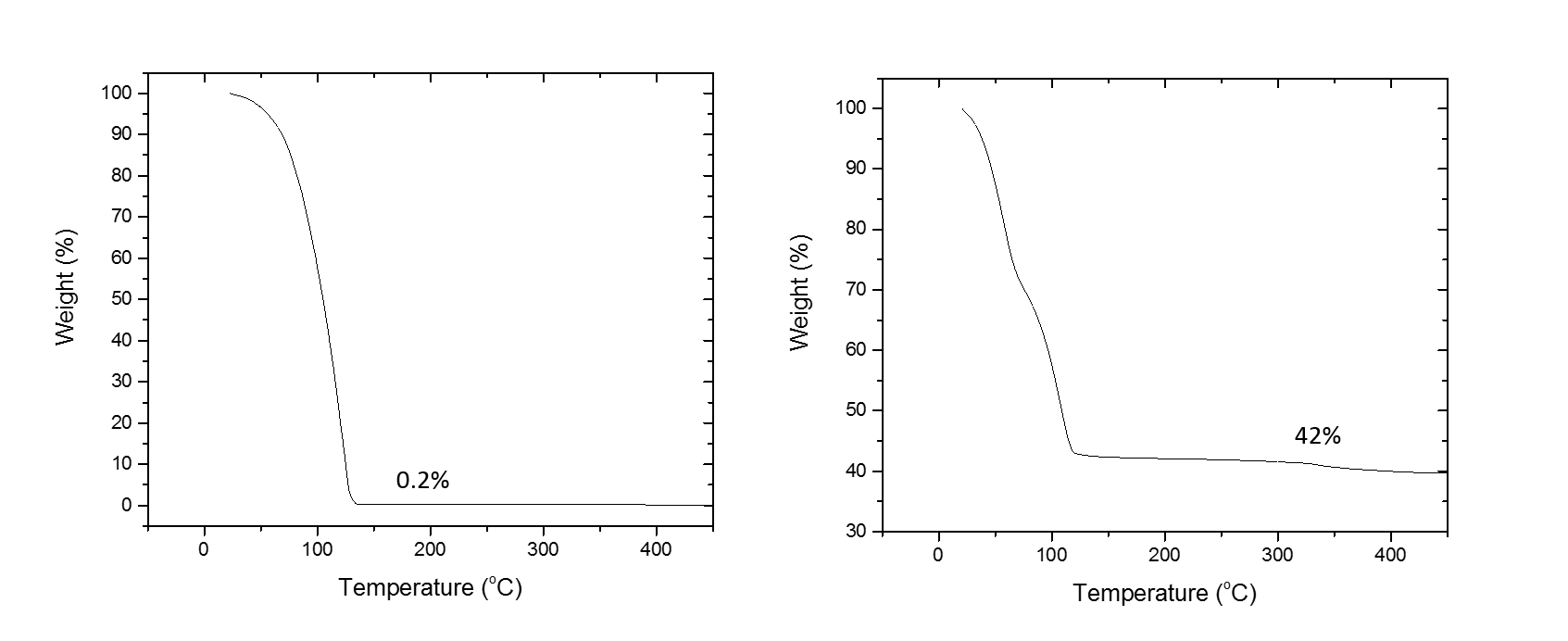

Supplement: Additional file 1: — Figure S1. Thermogravimetric analysis (TGA) in nitrogen of (a) CNT paste (CNT wt.% ~0.2) and (b) AgNPs ink (AgNPs wt.% ~42). (TIF 130 kb) [file 11671_2016_1626_MOESM1_ESM.tif]
